# Multiple Organ Dysfunction Syndrome and Pediatric Logistic Organ Dysfunction–2 Score in Pediatric Cerebral Malaria

**DOI:** 10.4269/ajtmh.22-0140

**Published:** 2022-09-06

**Authors:** Hunter Johnson, Madiha Raees, Evangelina Urbina, Jennifer Muszynski, Karl Seydel, Terrie Taylor, Nicole O’Brien

**Affiliations:** ^1^ Division of Pediatric Critical Care Medicine, Department of Pediatrics, Nationwide Children’s Hospital, The Ohio State University, Columbus, Ohio;; ^2^ Division of Pediatric Critical Care Medicine, Department of Pediatrics, Children’s Hospital of Philadelphia, Perelman School of Medicine at the University of Pennsylvania, Philadelphia, Pennsylvania;; ^3^ Division of Pediatric Critical Care Medicine, Department of Pediatrics, Hospital de Especialidades Pediatricas, Universidad Nacional Autónoma de México, Tuxtla Gutiérrez, Chiapas, México;; ^4^ Department of Osteopathic Medical Specialties, College of Osteopathic Medicine, Michigan State University, East Lansing, Michigan;; ^5^ Blantyre Malaria Project, University of Malawi College of Medicine, Blantyre, Malawi

## Abstract

Malaria resulted in an estimated 627,000 deaths in 2020, the majority of which occurred in children under 5 years of age. Cerebral malaria (CM) is a severe manifestation of the disease with case fatality rates of up to 40%. Autopsies in children with CM have demonstrated sequestration of *Plasmodium falciparum* parasites in the brain as well as multiple other organs. Thus, multiple organ dysfunction syndrome (MODS) may be present in pediatric patients with CM, but its frequency and association with mortality have not been evaluated. This is a retrospective study of data collected prospectively from children with CM admitted in Blantyre, Malawi. Physical examination findings and laboratory values necessary to calculate a Pediatric Logistic Organ Dysfunction–2 (PELOD-2) score, a validated method that quantifies organ dysfunction in critically ill children, were abstracted. A total of 145 patients were included. Mortality was 15% (*n* = 22). Ten patients (7%) had single organ dysfunction, 36 (25%) had two organs involved, 68 (47%) had dysfunction of three organs, and 31 (21%) patients had four organs affected. Beyond neurologic dysfunction, other organ systems involved included hematologic (77%), renal (61%), cardiovascular (44%), and respiratory (1%). The median PELOD-2 score on admission was 4 (interquartile range [IQR] = 3–6) in survivors and 6.5 (IQR = 5–10) in the nonsurvivors (*P* < 0.0001). Admission PELOD-2 score predicted mortality with an area under the curve of 0.75. MODS is widespread in pediatric patients with CM. Objectively identifying children with MODS, and therefore at an increased risk of mortality, may allow for the allocation of limited resources.

## INTRODUCTION

In 2020, malaria resulted in approximately 627,000 deaths worldwide with 96% occurring in Africa, primarily in children less than 5 years of age.^1^ Cerebral malaria (CM), defined as Blantyre Coma Score ≤ 2, *Plasmodium falciparum* parasitemia, and no other discernible cause of coma, is one of the most severe manifestations of malaria. Despite advances in both malaria prevention and treatment, mortality rates in children with CM continue to range from 15% to 40%, with neurological sequalae identified in up to 50% of survivors at the time of long-term follow-up.^2^^–^^8^

*P. falciparum* parasitized red blood cells can be found adherent to the microvasculature of many organs within its host.^9^^–^^11^ Although multiple organ dysfunction syndrome (MODS), presumably secondary to microvascular occlusion or widespread endothelial cell activation with subsequent inflammatory cascade initiation, is reported as a frequent complication of severe malaria in adults, it has not been well described in children.^12^ An estimated 33% to 85% of adult patients with *P. falciparum* malaria have MODS when using the Sequential Organ Failure Assessment score to define organ dysfunction.^13^^,^^14^ This is clinically significant due to the increased mortality rates reported in adult malaria patients with MODS (48.8%) compared with those with less than two organ systems involved (6.4%).^13^

In a study by Milner et al., autopsy results of 53 Malawian children with CM demonstrated significant sequestration of parasites within the brain, heart, lungs, spleen, stomach, small intestines, large intestines, and skin.^15^ Based on the parasite sequestration seen in multiple organs in this cohort, it is plausible that multiorgan dysfunction is also present in pediatric patients with CM. However, our current understanding of MODS in this patient population is lacking. In one study, Kochar et al. found that 48 of 79 (61%) pediatric patients with severe *P. falciparum* malaria had evidence of multiorgan involvement. Multiple organ dysfunction was present in 14 of the 17 patients (82%) with CM. In children with both CM and multiple organ dysfunction, case fatality rates were 14.3% compared with 5.9% in those with multiple organ dysfunction alone, but standard definitions of organ dysfunction were not used.^16^

The Pediatric Logistic Organ Dysfunction–2 (PELOD-2) score is a validated scoring system used to identify MODS and predict mortality in critically ill children (Table 1).^17^ In this retrospective study, we hypothesize that Malawian children with CM will have evidence of multiorgan failure based on their PELOD-2 admission scores. Furthermore, we hypothesize that patients with higher PELOD-2 scores on admission will have increased mortality rates. If these hypotheses are correct, quickly identifying children with MODS at the time of admission can assist with the determination of which patients are most likely to benefit from aggressive intervention with available resources.

**Table 1:** Pediatric Logistic Organ Dysfunction–2 Score^17^

Organ dysfunction and variables	Points by severity levels						
	0	1	2	3	4	5	6
Neurologic							
Glasgow Coma Score*	≥ 11	5–10			3–4		
Pupillary reaction	Both reactive					Both fixed	
Cardiovascular							
Lactatemia (mmol/L)	< 5.0	5.0–10.9			≥ 11		
Mean arterial pressure (mm Hg)							
Age (months)							
0 to < 1	≥ 46		31–45	17–30			≤ 16
1–11	≥ 55		39–54	25–38			≤ 24
12–23	≥ 60		44–59	31–43			≤ 30
24–59	≥ 62		46–61	32–44			≤ 31
60–143	≥ 65		49–64	36–48			≤ 35
≥ 144	≥ 67		52–66	38–51			≤ 37
Renal							
Creatinine (µmol/L)							
Age (months)							
0 to < 1			≥ 70				
1–11	≤ 22		≥ 23				
12–23	≤ 34		≥ 35				
24–59	≤ 50		≥ 51				
60–143	≤ 58		≥ 59				
≥ 144	≤ 92		≥ 93				
Respiratory							
PaO_2_ (mm Hg)/FiO_2_†	≥ 61		≤ 60				
PaCO_2_ (mm Hg)	≤ 58	59–94		≥ 95			
Invasive ventilation	No			Yes			
Hematologic							
WBC count (×10^9^/L)	> 2		≤ 2				
Platelets (×10^9^/L)	≥ 142	77–141	≤ 76				

PaO_2_ = partial pressure of oxygen; FiO_2_ = fraction of inspired oxygen; PaCO_2_ = partial pressure of carbon dioxide; WBC = white blood cell.

The following adjustments were made in this study: * Blantyre Coma Score (BCS) of 4–5 = 0 points, BCS of 1–3 = 1 point, BCS of 0 = 4 points; † SpO_2_/FiO_2_ ratio of ≥ 100 = 0 points and < 100 = 2 points.

## MATERIALS AND METHODS

This is a retrospective study of data collected prospectively from January 2019 to June 2021 at Queen Elizabeth Central Hospital (QECH) in Blantyre, Malawi, as an ancillary study to Treating Brain Swelling in Pediatric Cerebral Malaria (5U01AI126610-02). The study was approved by the ethics committee at Michigan State University and at the University of Malawi College of Medicine Research Ethics Committee. All subjects’ guardians provided both verbal and written informed consent before enrollment.

The parents or caregivers of children 6 months to 12 years of age who met the WHO case definition of cerebral malaria (Blantyre Coma Score [BCS] ≤ 2, peripheral parasitemia with *Plasmodium falciparum*, and no other discernable cause of encephalopathy) were approached for enrollment. Demographic data, vital signs, physical examination findings, and laboratory studies were collected at the time of admission. The patient’s blood pressure was taken on a supported upper extremity at heart level when the child was at their calmest state, using an electronic sphygmomanometer (Contec Medical Systems CONTEC08A, People’s Republic of China). The correct cuff size was selected based on the circumference of the arm with the bladder length equivalent to 80% to 100% of the circumference of the arm and the width at least 40% to ensure the most accurate reading.^18^

Finger-prick samples were analyzed to determine parasite species and density, packed-cell volume, and lactate concentration (Arkray Lactate Pro 2, Minneapolis, Minnesota). Venous blood was drawn to obtain a complete blood count, electrolyte analysis, and blood gas analysis (Beckman Coulter Life Sciences Coulter Counter, Indianapolis, Indiana; Abbott iSTAT, Princeton, New Jersey). All patients received treatment of severe malaria according to the Malawi Standard Treatment Guidelines, which included the administration of intravenous artesunate, treatment of fever and hypoglycemia (defined as blood glucose < 2.2 mmol/L or 40 mg/dL), and the use of anticonvulsants and antibiotics if indicated. No patients were on inotropes, intubated, or ventilated at the time of admission.

Paper charts of all eligible patients were reviewed. Data required for the calculation of each of the five subscores that comprise the PELOD-2 score were extracted for each patient. Criteria were met for organ dysfunction if the score was > 0 in any organ specific subscore. If more than one organ system had a score > 0, the patient was diagnosed with MODS. The total PELOD-2 score was calculated and recorded. For the neurologic subscore, BCS was used instead of the Glasgow Coma Score (GCS). A GCS was not obtained for any patient at the time of admission, but there was a documented BCS for each patient because it is more commonly used in children with CM. A BCS of 4 to 5 was scored as 0 points, 1 to 3 was scored as 1 point, and 0 was scored as 4 points.^19^ Creatinine values were converted from mg/dL to µmol/L and then scored accordingly based on the age-appropriate ranges listed in the PELOD-2 scoring table. SpO_2_/FiO_2_ ratio was used instead of the PaO_2_/FiO_2_ ratio as arterial blood gases are not routinely obtained as part of standard of care. A SpO_2_/FiO_2_ ratio of ≥ 100 was scored as 0 points and < 100 was scored as 2 points.^20^^,^^21^ No modifications were made to the hematologic or cardiovascular subscores. The number of affected organs was noted for each patient along with which organ systems were involved.

Values are reported as median (interquartile range) or *n* (%) as appropriate. Comparisons between survivors and nonsurvivors were analyzed using the Fisher’s exact test for categorical data and Mann–Whitney test for continuous data. Correlation between age and PELOD-2 score was evaluated using the Spearman rank test. Receiver operator characteristic (ROC) curve analysis was used to assess the performance of the PELOD-2 score to predict mortality. Two-sided *P* values < 0.05 were considered statistically significant throughout. Analyses were performed using GraphPad Prism version 9.00 for Windows (GraphPad Software, La Jolla, CA).

## RESULTS

**Figure 1.**
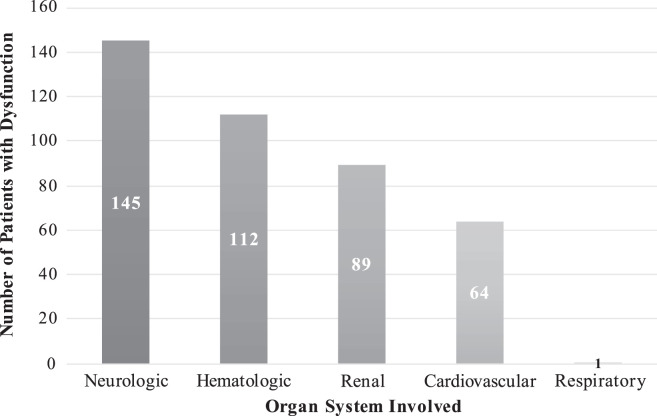
Organ systems affected in 145 pediatric patients with cerebral malaria.

The 145 patients consecutively admitted between January 29, 2019, and June 2, 2021, were reviewed. Basic demographics, vital signs, and admission laboratories are illustrated in Table 2. Ten (7%) patients had only neurologic dysfunction at the time of admission. One hundred and thirty-five patients (93%) met criteria for multiple organ dysfunction syndrome based on their PELOD-2 scores. Additional organ system involvement in our cohort included hematologic (77%), renal (61%), cardiovascular (44%), and respiratory (1%) (Figure 1).

**Table 2:** Demographics, laboratory investigations, and outcomes for the cohort (*N* = 145)

Variable	Value
Demographics	
Age, months, median (IQR)	54 (36.5–77.5)
Male sex, n (%)	68 (47)
Vital signs	
Temperature, °C, median, (IQR)	38.6 (38–39.4)
Oxygen saturation, %, median, (IQR)	97 (95–99)
Heart rate, beats/min, median, (IQR)	143 (129–160)
Tachycardia present, *n* (%)*	69 (48)
Respiratory rate,† breaths/min, median, (IQR)	36 (29–44)
Tachypnea present, *n* (%)*	52 (36)
MAP, mm Hg, median, (IQR)	77 (70–88)
Hypotension present, *n* (%)*	0 (0)
Laboratory investigations	
WBC count,‡ ×10^3^/µL, median (IQR)	9.5 (6.8–13.4)
> 2, *n* (%)	139 (99)
≤ 2, *n* (%)	2 (1)
Platelets,‡ ×10^3^/µL, median (IQR)	70 (37–124)
≥ 142, *n* (%)	29 (21)
77–141, *n* (%)	36 (25)
≤ 76, *n* (%)	76 (54)
Lactate, mmol/L, median (IQR)	3.9 (2.3–7.1)
< 5, *n* (%)	84 (58)
5.0–10.9, *n* (%)	40 (28)
≥ 11, *n* (%)	21 (14)
pH,^‖^ median (IQR)	7.4 (7.39–7.49)
Carbon dioxide,^‖^ mm Hg, median (IQR)	27 (23–31)
Sodium,§ mEq/L, median (IQR)	138 (134–142)
Potassium,‡ mEq/L, median (IQR)	4.5 (3.9–5.3)
Bicarbonate,§ mmol/L, median (IQR)	16 (11–18)
Creatinine,¶ µmol/L, median (IQR)	66 (50–92)
Bilirubin,# µmol/L, median (IQR)	18.8 (10.3–35.9)
Clinical features	
Retinopathy positive, *n* (%)	95 (66)
Blantyre coma score, *n* (%)	
0	20 (14)
1	52 (36)
2	73 (50)
Nonsurvivors, *n* (%)	22 (15)

IQR = interquartile range; MAP = mean arterial pressure; mEq = milliequivalent; WBC = white blood cell.

* Normative values based on the WHO’s Guidelines for the Management of Common Childhood Illnesses.^22^

† *N* = 144.

‡ *N* = 141.

§ *N* = 142.

^‖ ^*N* = 83.

¶ *N* = 130.

# *N* = 95.

**Figure 2.**
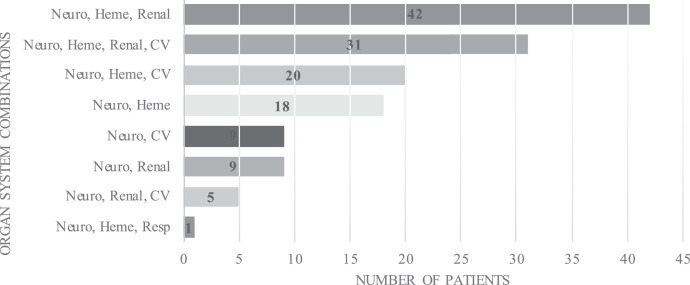
Combinations of dysfunctional organ systems in 135 pediatric cerebral malaria patients with multiple organ dysfunction syndrome. Neuro = neurologic; Heme = hematologic; CV = cardiovascular; Resp = respiratory.

Thirty-six patients (25%) had two organs involved, 68 (47%) had dysfunction of three organs, and the remaining 31 (21%) patients had four organs affected. There was no significant difference between the number of organ systems involved in survivors and nonsurvivors (Table 3). The different combinations of organs involved in the cohort are shown in Figure 2.

**Table 3:** Comparing pediatric cerebral malaria survivors versus nonsurvivors

Characteristic	All (*N* = 145)	Survivors (*N* = 123)	Nonsurvivors (*N* = 22)	*P* value
Age, months, median (IQR)	54 (36.5–77.5)	56 (39–79)	37.5 (25–55)	**0.009**
Male sex, *n* (%)	68 (47)	57 (46)	11 (50)	0.8
Retinopathy positive, *n* (%)	95 (66)	79 (64)	16 (73)	0.63
Number of organ dysfunctions, median (IQR)	3 (2–3)	3 (2–3)	3 (2–4)	0.2
Neurologic dysfunction, *n* (%)	145 (100)	123 (100)	22 (100)	1
Respiratory dysfunction, *n* (%)	1 (0.6)	1 (0.8)	0 (0)	1
Cardiovascular dysfunction, *n* (%)	64 (44)	50 (41)	14 (64)	0.06
Renal dysfunction, *n* (%)	89 (61)	75 (61)	14 (64)	1
Hematologic dysfunction, *n* (%)	112 (77)	95 (77)	17 (77)	1
PELOD-2 score, median (IQR)	5 (3–6)	4 (3–6)	6.5 (5–10)	**0.0001**
PELOD-2 score excluding neuro, median (IQR)	3 (2–5)	3 (2–4)	4.5 (2–7)	**0.04**
Neurologic PELOD-2 score, median (IQR)		1 (1–1)	4 (1–4)	**< 0.0001**
Respiratory PELOD-2 score, median (IQR)		0 (0–0)	0 (0–0)	1
Cardiovascular PELOD-2 score, median (IQR)		0 (0–1)	4 (1–6)	**0.015**
Renal PELOD-2 score, median (IQR)		2 (2–2)	2 (2–2)	1
Hematologic PELOD-2 score, median (IQR)		2 (2–2)	2 (2–4)	0.14

IQR = interquartile range; neuro = neurologic; PELOD-2 = Pediatric Logistic Organ Dysfunction-2.

Overall mortality for the cohort was 15% (*n* = 22). The median PELOD-2 score on admission was 4 (interquartile range [IQR] = 3–6) in those who survived to discharge and 6.5 (IQR = 5–10) in the nonsurvivors (*P* < 0.0001; Table 3). When the neuro subscore was removed, the total PELOD-2 score remained higher in the nonsurvivors (*P* = 0.04). Ninety-five subjects had evidence of retinopathy. Presence of retinopathy was not associated with PELOD-2 score. For patients with retinopathy, median PELOD-2 score was 5 (IQR = 3–6) versus 5 (IQR = 3–6.25) for those who were retinopathy negative (*P* = 0.62). Retinopathy was not associated with mortality (16.8% versus 12%, *P* = 0.63). Although age was associated with mortality in this cohort, age was not associated with PELOD-2 score (*r* = –0.08, *P* = 0.37). As a sensitivity analysis, we performed multivariable logistic regression with age and PELOD-2 score included in the model. When accounting for age, a higher PELOD-2 score remained associated with decreased odds of survival (aOR 0.73; 95% confidence interval [CI]: 0.61–0.86).

**Figure 3.**
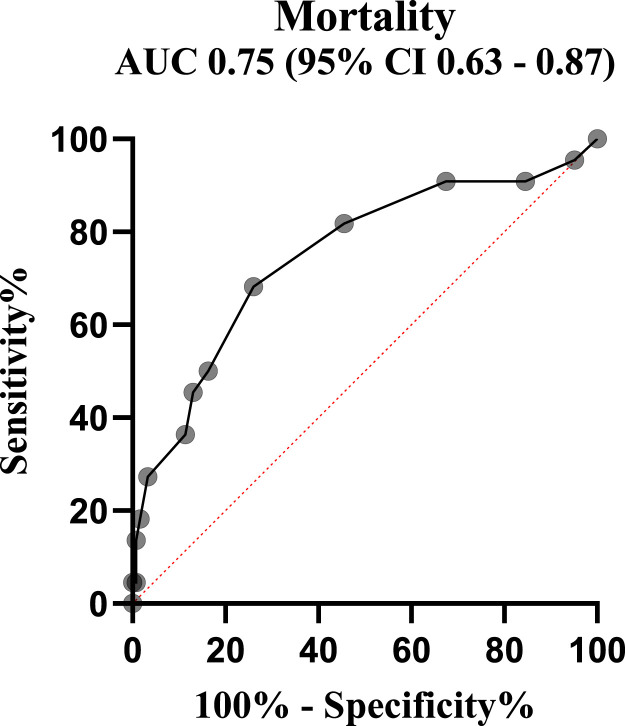
Receiver operating curve showing ability of Pediatric Logistic Organ Dysfunction–2 score to predict mortality. CI = confidence interval. This figure appears in color at www.ajtmh.org.

An ROC analysis showed that admission PELOD-2 score predicted mortality with an area under the curve of 0.75. (Figure 3). The optimal cut point as defined by Youden’s J statistic was a PELOD-2 score > 5.5. Sensitivity and specificity for this cut point are 68% (95% CI: 47–84) and 74% (95% CI: 66–81), respectively.

## DISCUSSION

Malaria continues to result in significant morbidity and mortality with more than 90% of severe and fatal malaria affecting children less than 5 years of age in sub-Saharan Africa.^23^^–^^27^ CM remains the most severe complication of *P. falciparum* infection. Multisystem, multiorgan involvement has been well described in adults with malaria, including those with CM, with significant increases in mortality in those diagnosed with MODS.^12^^,^^14^^,^^28^^,^^29^ In adults with only neurologic dysfunction or “pure CM,” mortality rates have been reported as low as 8% compared with nearly 50% seen in those with additional organ dysfunction.^11^ Despite autopsy studies showing evidence of multiorgan sequestration of malaria parasites in pediatric patients with CM, there are limited data available that evaluate the incidence of MODS and its effect on outcomes in these patients.^3^^,^^4^^,^^15^^,^^16^^,^^30^ Due to the paucity of evidence on this question, we applied the PELOD-2 score, a scoring system widely used and accepted in critically ill pediatric patients to identify MODS and evaluate the effect of this complication on mortality, to pediatric CM patients. Notably, other predictive scoring systems have been created for use in resource limited settings such as the Lambaréné Organ Dysfunction Score, the Pediatric Early Death Index for Africa, and Signs of Inflammation in Children that Kill.^31^^–^^34^ While these scoring systems have been shown to predict mortality, none are validated for the diagnosis of multiple organ dysfunction syndrome, and all have limitations in this patient population. For example, the Lambaréné Organ Dysfunction Score is comprised of only three clinical variables (coma, prostration, and deep breathing) which allows for rapid scoring.^31^ However, two of the three variables are related to the patient’s neurologic status limiting its utility in CM patients.

In our cohort of 145 pediatric patients with CM, using a PELOD-2 score, we found evidence of MODS (two or more organ systems with evidence of dysfunction) in 135 patients (93%) at the time of admission. Neurologic dysfunction was present in all by definition. The hematologic system was the second most affected organ system, with dysfunction defined as leukopenia or thrombocytopenia. This finding is not surprising because thrombocytopenia is a well-recognized, common complication of malaria with several proposed underlying mechanisms including immune-mediated destruction, splenic sequestration, bone marrow suppression, and consumption in the microvasculature.^35^^,^^36^

Renal dysfunction, defined as elevated creatinine level for age, was the third most common type of organ dysfunction in our cohort. Hypovolemia with resultant low renal blood flow and secondary renal ischemia, tubular necrosis due to hemoglobinuria and cytokine-induced apoptosis, and myoglobinuria from muscle necrosis are proposed mechanisms of acute renal failure in patients with severe malaria.^13^^,^^37^^–^^39^ Renal failure is a commonly reported complication of severe malaria in adults but has previously been reported as rare in children, although this may be changing.^23^^,^^40^ With the application of consensus definitions for acute kidney injury (AKI) in children, AKI is now being reported in 24% to 59% of pediatric patients with severe malaria.^41^^–^^45^ Additionally, two recent studies have demonstrated an increased risk of AKI in African children with severe malaria who are < 2 years of age.^43^^,^^46^ Our study is consistent with these findings because all patients in our cohort who were < 2 years old met the PELOD-2 criteria for renal dysfunction. AKI is a known independent risk factor for mortality in malaria, which may be one plausible explanation of why we saw increased mortality in younger children in this cohort.^40^^,^^47^^–^^54^

Lastly, almost half of our cohort had cardiovascular dysfunction defined as elevated lactate levels, hypotension, or both. On the basis of prior studies, we believe the elevated lactate level (≥ 5 mmol/L) found in > 40% of our patients was likely multifactorial and a combination of the following: 1) poor perfusion with resultant tissue anoxia and subsequent anaerobic glycolysis, 2) decreased clearance of lactate in the setting of liver dysfunction, and 3) the release of lactate by parasitized erythrocytes.^23^^,^^55^ Eight patients (5.5%) had hypotension at the time of admission, which may have been related to hypovolemia in the setting of dehydration, a concurrent bacterial infection with resultant septic shock, or myocardial dysfunction from parasitized red blood cells in the myocardial vessels.^23^^,^^56^

The presence of MODS was associated with increased mortality in our cohort of patients. Additionally, nonsurvivors were found to have higher PELOD-2 scores at the time of admission. There was no significant difference in the number of organ systems involved between survivors and nonsurvivors, suggesting that the association with outcome may be related to more severe involvement of the affected organs. The increase in mortality in pediatric CM patients in the presence of MODS illustrates the need for improved awareness of MODS so management strategies for these patients can include appropriate multiorgan, multisystem support. If a child with CM has cardiovascular dysfunction, maintaining appropriate cerebral perfusion pressure without pharmacologic assistance, particularly if intracranial hypertension is present, will be difficult.^57^ A CM patient with renal dysfunction may be at risk for worsening neurologic dysfunction due to associated uremic encephalopathy or may develop life-threatening electrolyte derangements. In this large cohort, multiple combinations of organ dysfunction were observed. We propose the need for further work to evaluate the effects of each combination on overall morbidity and mortality.

Our work has several limitations. This is a retrospective study that took place in a resource-limited setting. Although our center may have access to resources not widely available in other malaria-endemic regions, compared with higher income countries, healthcare resources are lacking. There were instances of missing data attributed to times when testing strips or laboratory reagents were not available. In these cases, scoring missing data as normal may have resulted in underestimating the overall frequency as well as the severity of MODS in multiple patients. We may have underestimated the presence of renal failure in our cohort because 15 patients (10%) were missing a creatinine at the time of admission. We propose the need for further studies investigating the frequency of renal failure in pediatric patients with severe malaria.

Lastly, the potential limitations of the PELOD-2 scoring system itself must be considered when used in this population. For example, respiratory dysfunction in the PELOD-2 score is partially determined by the presence of mechanical ventilation, which is not a common intervention where malaria is endemic. Additionally, the ability to obtain an arterial blood gas is extremely rare in these settings, limiting the feasibility of calculating a PaO_2_/FiO_2_ ratio. Despite other studies reporting the clinical recognition of respiratory distress in up to 40% of pediatric patients with severe malaria, only 1 out of 145 patients in our work met PELOD-2 criteria for respiratory dysfunction.^58^ Furthermore, due to parasite metabolism and sequestration, hyperlactatemia and thrombocytopenia are common in patients with severe malaria and may not represent dysfunction of the cardiac and hematologic systems respectively.^23^ Adapted PELOD-2 scoring that uses alternative indicators of respiratory failure, shock, and disseminated intravascular coagulation in children with severe malaria is needed.

Multiple organ dysfunction is prevalent in pediatric patients with CM. MODS is associated with increased mortality in this population, and admission PELOD-2 scores may objectively identify children with CM at highest risk of mortality allowing for the allocation of limited resources. Given the frequency of MODS we identified, recognizing additional organ systems involved is essential when providing patient care and making management decisions.
